# Blackcurrant (*Ribes nigrum*) Extract Exerts an Anti-Inflammatory Action by Modulating Macrophage Phenotypes

**DOI:** 10.3390/nu11050975

**Published:** 2019-04-28

**Authors:** Yoojin Lee, Ji-Young Lee

**Affiliations:** Department of Nutritional Sciences, University of Connecticut, Storrs, CT 06269, USA; yoojin.lee@uconn.edu

**Keywords:** blackcurrant, macrophage, macrophage polarization, macrophage phenotype, polyphenol

## Abstract

Macrophages are polarized into different phenotypes depending on tissue microenvironment where they reside. In obesity-associated inflammation, M1-type macrophages are predominant in the inflamed tissue, exerting pro-inflammatory responses. Our previous studies demonstrate that blackcurrant consumption attenuates hepatic inflammation and lipopolysaccharide-stimulated inflammatory responses of splenocytes in obese mice. In this study, we determined whether blackcurrant modulates macrophage phenotypes to exert its anti-inflammatory action. Mouse bone marrow-derived macrophages (BMDM) and human THP-1 macrophages were polarized into M1 macrophages in the presence or absence of blackcurrant extract (BCE). BCE repressed M1 polarization of both murine and human macrophages. Also, to gain insight into the role of blackcurrant metabolites produced in vivo in the regulation of macrophage phenotypes, BMDM were treated with serum obtained from lean or obese mice fed blackcurrant. While serum from lean mice fed blackcurrant did not exert either anti-inflammatory actions or suppressive effects on M1 polarization, serum from obese mice fed blackcurrant reduced the expression of pro-inflammatory genes in BMDM. Our data demonstrate that BCE suppresses M1 polarization, with reduced pro-inflammatory responses. Moreover, this study suggests that blackcurrant metabolites may not exert their anti-inflammatory effect directly by altering macrophage phenotypes, but possibly by inhibiting the production of obesity-associated inflammatory factors.

## 1. Introduction

Macrophages are a heterogeneous population of immune cells that are distributed in all organs and play a crucial role in tissue homeostasis and host defense [1]. Macrophages are functionally plastic in response to inflammatory cues from microbial products, damaged tissues, and activated lymphocytes [2,3]. The functional change or adaptation of macrophages in response to stimuli is referred to as macrophage polarization [4]. Polarized macrophages are broadly categorized into classically activated M1 macrophages and alternatively activated M2 macrophages [5]. Typical stimuli that induce M1 polarization are interferon γ (IFNγ), tumor necrosis factor α (TNFα), and lipopolysaccharide (LPS), while interleukin-4 (IL-4), IL-10, and IL-13 are known to stimulate M2 polarization [5,6]. M1 macrophages produce pro-inflammatory cytokines, e.g., TNF, IL-1β, IL-12, and IL-6, and reactive oxygen species (ROS) [5,6]. On the other hand, M2 macrophages are specialized to produce immune-modulatory and reparative mediators such as IL-10, transforming growth factor β (TGFβ), IL-4, and IL-13, and to clear apoptotic cells due to their high phagocytosis capacity [7].

In obesity-associated inflammation, M1 macrophages are predominant in the inflamed tissue, producing ROS, nitric oxide (NO), and pro-inflammatory cytokines [8], which aggravate inflammation and damage tissue. Specifically, studies have reported that M1 macrophages in the liver and adipose tissue are increased in obesity [9,10,11,12]. Therefore, it is beneficial to inhibit macrophage polarization into M1 or to shift macrophage phenotype toward anti-inflammatory and pro-resolving M2 macrophages to prevent obesity-associated inflammation [13].

Blackcurrant (*Ribes nigrum*) is a type of berries originated from Northern Asia and Europe, and started to gain its popularity in the U.S. from the early 2000s [14]. Blackcurrant contains high phenolic compounds the majority of which are anthocyanins [14]. Specifically, we previously reported that the major anthocyanins in blackcurrant are delphinidin-3-rutinoside, cyanidin-3-rutinoside, delphinidin-3-glucoside, and cyanidin-3-glucoside [15]. The anthocyanins in blackcurrant contribute to its antioxidant, anti-inflammatory, and anti-microbial properties [14,15,16,17,18].

Our previous studies demonstrated that polyphenol-rich blackcurrant extract (BCE) prevented inflammation in the liver and adipose tissue of diet-induced obesity (DIO) mice [19,20]. Furthermore, we have shown increases in the hepatic expression of M1 macrophage markers, which was significantly attenuated by blackcurrant in DIO mice [12]. In the present study, we further investigated whether blackcurrant can modulate macrophage phenotypes to exert its anti-inflammatory actions with a primary focus on the role of blackcurrant in the regulation of macrophage polarization in vitro, utilizing both mouse and human macrophages. 

## 2. Materials and Methods

### 2.1. Animal Care and Diet

Seven-week-old male C57BL/6J mice (Jackson Laboratory, Bar Harbor, ME, USA) were randomly assigned into four groups: a low fat control (LF; 13% energy from fat, *n* = 7), a LF with blackcurrant (LF-BC; 6% of freeze-dried, whole blackcurrant powder by weight, *n* = 7), an obesogenic high fat/high sucrose control (HF; 57%/28% energy from fat/sucrose, *n* = 14), or a HF containing 6% blackcurrant powder (HF-BC, *n* = 15), as we previously described [12]. As C57BL/6J mice are prone to develop obesity [21], mice fed a LF or LF-BC were on the experimental diets for one week to avoid any compounding effects of obesity-induced pro-inflammatory mediators in the circulation. HF or HF-BC-fed mice were on the experimental diets for 22–23 weeks for serum collection. Serum samples were collected by cardiac puncture or from the lateral tail vein, followed by incubation for 30 min at room temperature and centrifugation at 2,000 × g at 4 °C for 10 min for clot removal. Serum was obtained during a feeding cycle to capture circulating blackcurrant metabolites. Mice were housed in a controlled environment under a 12-h light/dark cycle with free access to food and water. All procedures were approved by the Institutional Animal Care and Use Committee of the University of Connecticut.

### 2.2. Cell Culture and Treatments

Murine RAW 264.7 macrophages and human THP-1 monocytes were purchased from ATCC (Manassas, VA, USA). The cells were maintained in RPMI-1640 supplemented with 10% fetal bovine serum, 2 mmol/L L-glutamine, 1x vitamin mix, 100 U/mL penicillin, and 100 µg/mL streptomycin. THP-1 macrophages were prepared by differentiation of THP-1 monocytes using 10 ng/mL of phorbol 12-myristate 13-acetate (PMA) for 24 h, followed by 24 h of resting in a regular growth medium without PMA. Mouse bone marrow-derived macrophages (BMDM) were differentiated from bone marrow cells isolated from the tibia and femur of C57BL/6J mice as we previously described [22]. All cells were cultured in a humidified incubator at 37 °C with 5% CO_2_. For experiments, RAW macrophages were pre-treated with 0, 25, 50, 75, or 100 μg/mL of BCE (Artemis International, Fort Wayne, IN, USA) for 24 h, and then stimulated with 100 ng/mL of LPS for 6 h. BMDM were treated with 15% serum collected from mice fed one of the experimental diets for 18 h. For polarization experiments, BMDM were polarized into M1 macrophages by IFNγ and TNFα (10 ng/mL each; R&D Systems, Minneapolis, MN, USA) in the absence or presence of 50 μg/mL of BCE or 15% mouse serum for 24 h. A set of polarized BMDM was stimulated with 10 ng/mL of LPS for an additional 6 h. THP-1 macrophages were polarized into M1 using 25 ng/mL each of IFNγ and TNFα for 24 h with or without 50 μg/mL of BCE. All experiments were conducted at least 2–3 times in triplicates each time.

### 2.3. Cytokine Secretion Analysis

After 24 h of polarization of THP-1 macrophages, 500 µL of cell culture supernatant was used to characterize secreted cytokines using a Proteome Profiler Human Cytokine Array (R&D Systems) according to the manufacturer’s instruction.

### 2.4. Reverse Transcription and Quantitative Real-Time PCR (qRT-PCR)

Total RNA was isolated from cells using TRIzol reagent (Invitrogen, Grand Island, NY, USA) and reverse transcribed as previously described [23]. qRT-PCR analysis was performed using the SYBR Green procedure and CFX96TM real-time PCR detection system (BioRad, Hercules, CA, USA). Data were analyzed by the 2^−ΔΔCT^ method. Ribosomal protein lateral stalk subunit P0 (RPLP0) was used as a reference gene for data normalization. Primer sequences will be available upon request.

### 2.5. Statistical Analysis

One-way analysis of variance and Tukey’s post hoc analysis or unpaired *t*-test where appropriate were performed using GraphPad Prism 6 (GraphPad Software, La Jolla, CA, USA). P values less than 0.05 were considered significant, and all values are expressed as mean ± standard error of the mean (SEM).

## 3. Results

### 3.1. Repression of LPS Stimulated-Inflammatory Responses by BCE in RAW 264.7 Macrophages

**Figure 1:**
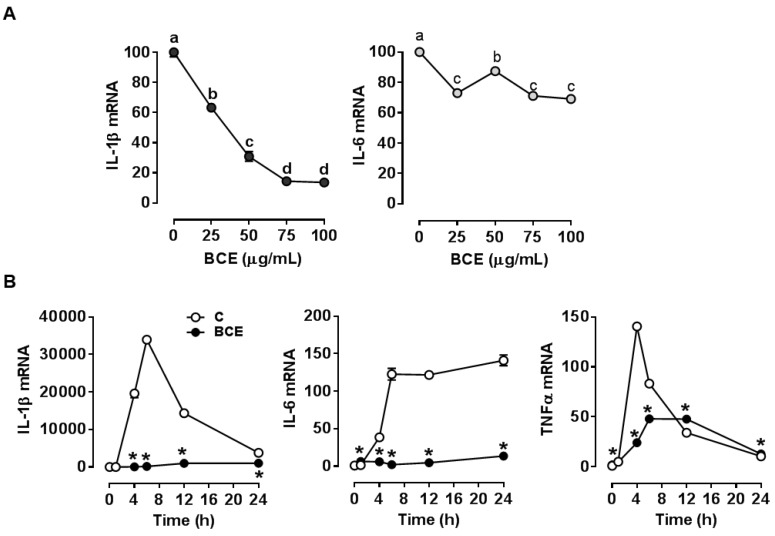
Effect of blackcurrant extract (BCE) on the expression of inflammatory genes in RAW 264.7 macrophages. (**A**) mRNA levels of interleukin-1β (IL-1β) and IL-6 in RAW 264.7 macrophages. Cells were pre-treated with 0 (control; C), 25, 50, 75, or 100 µg/mL of BCE for 24 h, and then stimulated with 100 ng/mL of lipopolysaccharide (LPS) for 6 h. (**B**) mRNA levels of IL-1β, IL-6, and tumor necrosis factor α (TNFα) in RAW 264.7 macrophages. Macrophages were pre-treated with 0 (control; C) or 50 µg/mL of BCE for 24 h and then stimulated with 100 ng/mL of LPS for 0, 1, 4, 6, 12, or 24 h. Data were normalized by no LPS control. Data are shown as mean ± SEM. Different letters show significant differences (*p* < 0.05). *, *p* < 0.05 vs. control.

In our previous study, we found blackcurrant consumption attenuated obesity-associated inflammation in the liver and LPS-stimulated inflammatory responses of splenocytes in HF diet-fed C57BL/6 mice [12]. As macrophages play a major role in inflammation, we determined whether blackcurrant exerts its anti-inflammatory actions via suppressing inflammatory responses in macrophages using polyphenol-rich BCE. BCE treatment decreased IL-1β and IL-6 mRNA levels upon LPS stimulation in RAW 264.7 macrophages (Figure 1A). Also, RAW 264.7 macrophages pre-treated with BCE attenuated mRNA abundance of IL-1β and IL-6 throughout 24 h of LPS stimulation, while TNFα mRNA was reduced by BCE until 6 h (Figure 1B).

### 3.2. Suppression of Macrophage Polarization into M1 by BCE in BMDM

**Figure 2:**
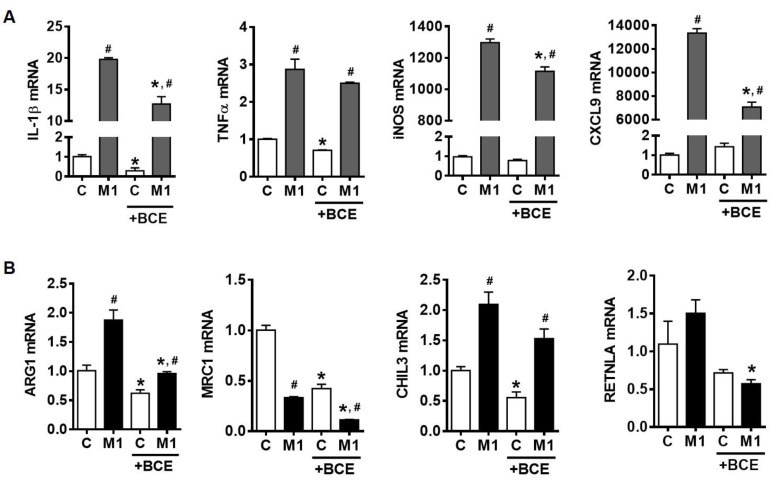
Expression of M1 and M2 marker genes in mouse bone marrow-derived macrophages (BMDM). BMDM were polarized to M1 using interferon γ (IFNγ) and tumor necrosis factor α (TNFα) (10 ng/mL each) with or without 50 µg/mL of blackcurrant extract (BCE) for 24 h. (**A**) The expression of M1 markers. (**B**) The expression of M2 markers. Data are shown as mean ± SEM. *, *p* < 0.05 vs. respective no BCE control. #, *p* < 0.05 vs. control within no or BCE treatment.

Increases in total macrophage numbers and the population of pro-inflammatory M1 macrophages in adipose tissue and the liver have been reported as a hallmark of obesity-associated chronic inflammation [9,10,11,12]. As LPS stimulated-inflammatory responses are closely related to M1 polarization [6], we determined whether the anti-inflammatory actions of BCE in macrophages could be attributable to its ability to suppress macrophage polarization to M1. BMDM were polarized into M1 macrophages using IFNγ and TNFα in the presence or absence of BCE. The expression of M1 macrophage markers, such as IL-1β, inducible NO synthase (iNOS), C-X-C motif ligand 9 (CXCL9), and TNFα, was induced when BMDM were polarized into M1. Importantly, the induction of IL-1β, iNOS, and CXCL9 expression was significantly reduced by BCE, while TNFα mRNA was not altered (Figure 2A). Furthermore, evidence suggests that a shift of the macrophage population to M2-type is protective against obesity-associated inflammation [11,24]. mRNA levels of M2 markers, including arginase (ARG1) and chitinase-like 3 (CHIL3), were increased in M1 macrophages, while mannose receptor C-type 1 (MRC1) mRNA was reduced, and resistin-like alpha (RETNLA) mRNA was not altered (Figure 2B). However, BCE attenuated the expression of ARG1 and RETNLA, and further decreased MRC1 expression.

### 3.3. Characterization of M1 Polarization in Human THP-1 Macrophages and the Role of BCE in Macrophage M1 Polarization

**Figure 3:**
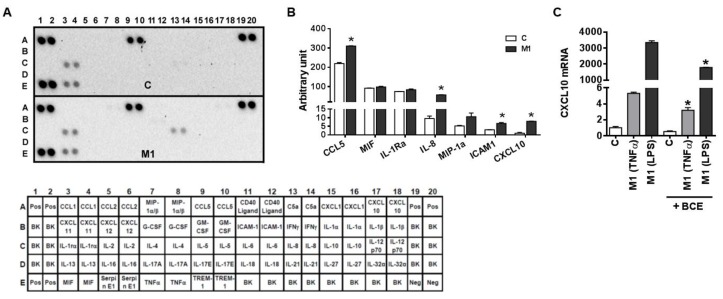
Characterization of cytokine secretion in human THP-1 macrophages. THP-1 monocytes were differentiated into macrophages in the presence of phorbol 12-myristate 13-acetate (PMA, 10 ng/mL) for 24 h. Subsequently, cells were incubated in regular growth medium without PMA for 24 h for resting, after which they were polarized into M1 using interferon γ (IFNγ) and tumor necrosis factor α (TNFα) or IFNγ and lipopolysaccharide (LPS) (25 ng/mL each). (**A**) Human cytokine array. Cell culture media from control (C, not treated with cytokines) or M1 macrophages (IFNγ and TNFα) were used to detect cytokines secreted from cells using a human cytokine array. Cytokine array layout is shown in the bottom. (**B**) Quantification of cytokines from the cytokine array. The intensity for each cytokine was normalized by positive controls for each blot. *, *p* < 0.05 vs. control. (**C**) The expression of C-X-C motif ligand 10 (CXCL10) in polarized THP-1 macrophages. Blackcurrant extract (BCE, 50 µg/mL) or control was treated during 24 h of polarization. Data are shown as mean ± SEM. *, *p* < 0.05 vs. respective no BCE control.

It is noted that human and murine macrophages have different phenotypical characteristics, such as NO metabolism and marker genes [25]. Therefore, we determined whether BCE exerts the repressive effect on macrophage polarization into M1 in human THP-1 macrophages. THP-1 macrophages were polarized into M1 macrophages using IFNγ and TNFα, and cytokines in cell culture medium were determined using a human cytokine array (Figure 3A). Macrophage polarization into M1 increased the secretion of C-C motif chemokine ligand 5 (CCL5), IL-8, intercellular adhesion molecule 1 (ICAM1), and CXCL10 in THP-1 macrophages, while the secretion of macrophage migration inhibitory factor (MIF), IL-1 receptor antagonist (IL-1Ra), and macrophage inflammatory protein 1 alpha (MIP-1a) was unchanged by M1 polarization (Figure 3B). Furthermore, THP-1 macrophages were polarized into M1 using two combinations of stimuli, i.e., IFNγ + TNFα or IFNγ + LPS, as LPS is also considered as a major stimulus for M1 polarization. The expression of M1 marker, CXCL10, was significantly higher in macrophages stimulated by IFNγ and LPS than those polarized using IFNγ and TNFα (Figure 3C). Importantly, the CXCL10 mRNA abundance was significantly reduced by BCE in M1 macrophages polarized by both stimuli combinations (Figure 3C).

### 3.4. Attenuation of Inflammatory Responses to LPS Stimulation in both Resting and Polarized BMDM

**Figure 4:**
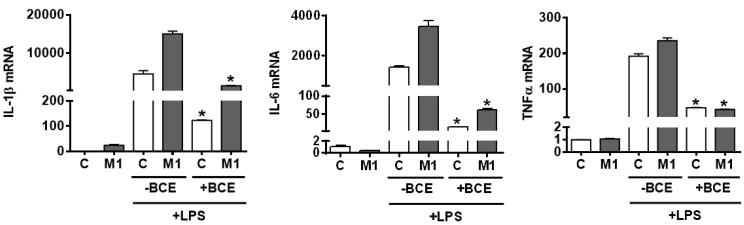
Expression of pro-inflammatory genes upon lipopolysaccharide (LPS) stimulation in M1 polarized mouse bone marrow-derived macrophages (BMDM). BMDM were polarized to M1 using interferon γ (IFNγ) and tumor necrosis factor α (TNFα) (10 ng/mL each) with or without 50 µg/mL of blackcurrant extract (BCE) for 24 h, followed by stimulation with 10 ng/mL of LPS for 3 h. Data are shown as mean ± SEM. *, *p* < 0.05 vs. respective no BCE control.

As we observed BCE reduced M1 polarization in both murine and human macrophages, we further investigated whether the effect of BCE on M1 polarization leads to different inflammatory responses to LPS stimulation. BMDM were polarized into M1 with or without BCE, after which they were stimulated with LPS. The LPS-induced expression of pro-inflammatory genes, including IL-1β, IL-6, and TNFα, was markedly attenuated by BCE in M1 macrophages (Figure 4). The reduction in the expression of IL-1β, IL-6, and TNFα was also observed in BCE-treated control group (Figure 4).

### 3.5. Effect of Mouse Serum in BMDM and Its Polarization

**Figure 5:**
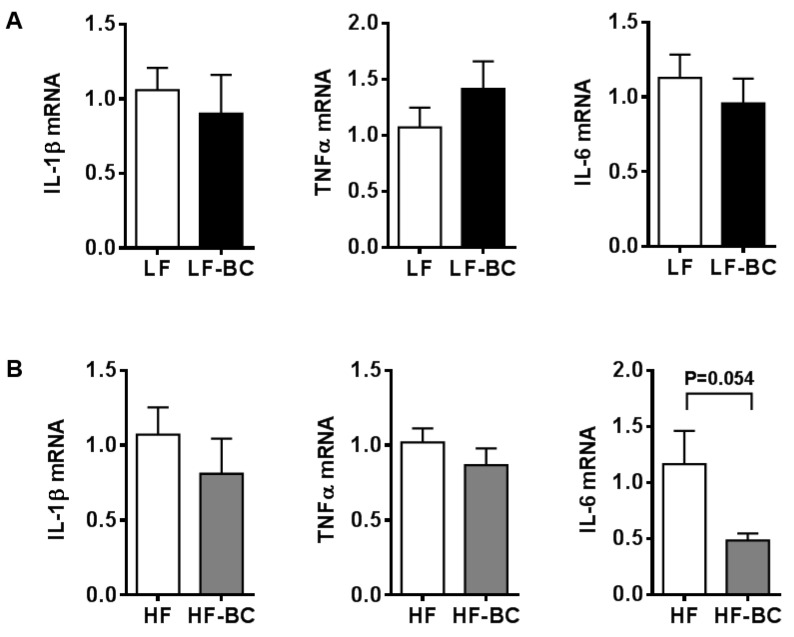
Expression of pro-inflammatory genes in mouse bone marrow-derived macrophages (BMDM) treated with serum from mice fed blackcurrant. (**A**) Mouse serum was collected during the feeding cycle from mice fed a low fat control (LF) or a LF with blackcurrant (LF-BC, LF supplemented with 6% of freeze-dried whole blackcurrant powder by weight) for one week (*n* = 7 per group). BMDM were treated with 15% serum for 18 h for gene analysis. (**B**) Mouse serum was collected during the feeding cycle from mice fed an obesogenic high fat/high sucrose control (HF) or a HF with 6% blackcurrant (HF-BC) for 22–23 week (*n* = 14 and 15, respectively). Serum samples from three mice were pooled and BMDM were treated with 15% the serum for 18 h. Data are shown as mean ± SEM.

**Figure 6:**
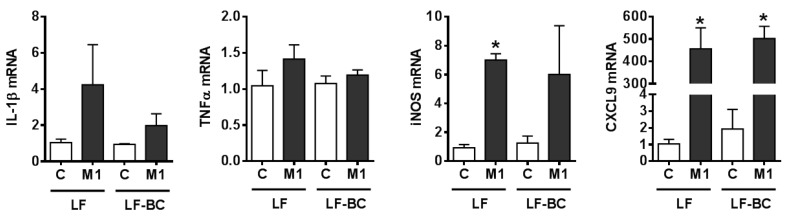
Expression of M1 marker genes in mouse bone marrow-derived macrophages (BMDM) treated with serum from mice fed a low fat control (LF) or a LF with blackcurrant (LF-BC, LF supplemented with 6% of freeze-dried whole blackcurrant powder by weight) for one week (*n* = 7 per group). Serum samples from two or three mice were pooled and BMDM were polarized into M1 using interferon γ (IFNγ) and tumor necrosis factor α (TNFα) (10 ng/mL each) for 24 h in the presence of 15% mouse serum. Data are shown as mean ± SEM. *, *p* < 0.05 vs. respective control.

The major components of BCE are anthocyanins, which are highly metabolized in vivo. Therefore, BMDM were treated with serum collected from mice fed a diet containing 6% whole blackcurrant (w/w). Blood was collected during the feeding cycle to capture blackcurrant metabolites in serum. Furthermore, we had two different sets of mice, i.e., LF and LF-BC, or HF and HF-BC. LF or LF-BC-fed mice were on the experimental diets only for one week to prevent any confounding effects of obesity-induced pro-inflammatory mediators in the circulation. On the other hand, mice fed a HF or HF-BC were on the experimental diets for 22–23 weeks. Treatment of serum from mice fed a LF-BC did not change the expression of pro-inflammatory genes, i.e., IL-1β, TNFα, and IL-6, compared to that of LF control (Figure 5A). However, IL-6 mRNA was trending toward a decrease by HF-BC serum compared to that of HF control, with minimal changes in IL-1β and TNFα mRNA (Figure 5B). Furthermore, when serum from LF or LF-BC-fed mice was treated during BMDM polarization into M1 using IFNγ and TNFα, there was no significant difference in the expression of M1 markers between LF or LF-BC serum-treated BMDM (Figure 6).

## 4. Discussion

In our previous study, we have shown anti-inflammatory effects of whole blackcurrant consumption in the liver of DIO mice, attenuating the development of non-alcoholic fatty liver disease [12]. Importantly, we found blackcurrant supplementation reduced obesity-induced macrophage infiltration into the liver with a decrease in the expression of M1 macrophage markers. While M1 macrophages are increased during obesity-associated chronic inflammation, a reduction in M1 population is shown to prevent chronic inflammation in the liver and adipose tissue [9,10,11,12,24]. Therefore, in the present study, we evaluated whether blackcurrant exerts its anti-inflammatory effect in macrophages via the modulation of macrophage phenotypes. Here, we demonstrated that BCE represses the polarization of M1 macrophages in both mouse and human macrophages, which is likely attributable to its anti-inflammatory effects in vivo. Furthermore, blackcurrant metabolites may not directly alter macrophage phenotypes, rather they may possibly modulate obesity-associated circulating pro-inflammatory factors, resulting in attenuated inflammatory responses in macrophages.

Macrophages are polarized into different phenotypes depending on types of stimuli in the microenvironment of tissues. Local immune milieu can be classified into T helper type 1 (Th1) and Th2, which are associated with differentiation of classically activated M1 and alternatively activated M2 macrophages, respectively [26]. Therefore, Th1 cytokines such as IFNγ and TNFα are commonly used as agents for M1 polarization [26]. The polarization of BMDM into M1 macrophages induced the expression of IL-1β, TNFα, CXCL9, and iNOS; however, BCE attenuated the induction, suggesting its repressive effect on macrophage M1 polarization. Suppressed M1 polarization led to diminished LPS-induced inflammatory responses in BMDM with less production of pro-inflammatory cytokines upon LPS stimulation than the control. Therefore, our data indicate that BCE represses M1 polarization in BMDM, which reduces inflammatory actions that could otherwise be triggered by various stimuli present in obesity-associated inflammation.

Certain characteristics of human and murine macrophages are known to be different [25,27]. For example, murine macrophages produce large amounts of NO and L-citrulline from L-arginine through the action of iNOS [25]. Also, murine macrophages produce tetrahydrobiopterin (BH4), which stabilizes iNOS. However, human macrophages do not have an iNOS activity or synthesize BH4 [28]. Also, murine M2 macrophages are characterized by consumption of L-arginine by arginase enzyme, whose activity is controversial in human macrophages [25]. Therefore, we confirmed whether the suppressive action of BCE in M1 polarization is also present in human macrophages. We found BCE treatment during M1 polarization decreased the expression of M1 marker, CXCL10, which was consistent with murine macrophages, suggesting the repressive action of BCE in M1 polarization. Therefore, our data demonstrated that the anti-inflammatory effect of BCE through the modulatory effect of macrophage phenotype is consistent in both murine and human macrophages.

Even though we observed BCE plays a role in the modulation of macrophage phenotypes, we acknowledge that BCE is a polyphenol-rich extract. Anthocyanins are known to be biotransformed in vivo and therefore they primarily exist as their metabolized forms—such as phenolic acids and aldehydes, and methyl, sulfate, and glucuronyl conjugates in the circulation [29]. To gain insight into the effect of blackcurrant consumption on the modulation of macrophage phenotypes in vivo, serum samples from lean and obese mice fed blackcurrant were collected during a feeding cycle to capture blackcurrant metabolites in the circulation. Serum from lean mice fed blackcurrant did not elicit either anti-inflammatory or suppressive effects on M1 polarization, suggesting that blackcurrant metabolites may not exert the anti-inflammatory effect in macrophages by directly altering macrophage phenotypes as does BCE. However, we cannot rule out the possibility that the amount of the metabolites in serum to which BMDM were exposed might not represent the similar levels of blackcurrant metabolites in vivo. On the other hand, BMDM treated with serum from obese mice fed blackcurrant showed less expression of pro-inflammatory genes. As the anti-inflammatory action of blackcurrant-fed mouse serum was only seen with HF group, it is presumed that the anti-inflammatory effect of serum from blackcurrant-fed obese mice is possibly through the reduction of circulating inflammatory mediators by blackcurrant consumption. Notably, increases in pro-inflammatory insults (e.g., C-reactive protein, IL-6, IL-8, TNFα, non-esterified fatty acids, ROS, and endotoxin) and decreases in anti-inflammatory factors (e.g., IL-10 and insulin-like growth factor) in blood have been reported in obese subjects [30,31]. As we did not observe either any increase in serum free fatty acids by HF diet or its reduction by blackcurrant consumption in obese mice [12], further studies are warranted to elucidate whether inflammation-modulating factors are altered in the serum of mice fed a HF-BC and whether the changes exert anti-inflammatory effects in macrophages.

Furthermore, the levels of circulating miRNAs such as miR-34a, miR-126, miR-146a, miR-150, miR-140-5p, miR-142-3p, miR-222, and miR-148a are altered in obese humans [32,33,34]. Indeed, we have reported a reduction in circulating miR-148a levels by blackcurrant consumption in obese mice [12], which may contribute to the anti-inflammatory action of blackcurrant, as miR-148a is suggested to promote M1 polarization [35]. Although analysis of blackcurrant metabolites in the circulation is beyond the scope of the present study, our result suggests that blackcurrant metabolites may not exert anti-inflammatory actions through the same mechanisms as does BCE. Instead, it is probable that in vivo anti-inflammatory effects of blackcurrant are likely mediated at least through the modulation of circulating obesity-associated inflammatory factors.

Although serum collected from blackcurrant-fed mice did not alter macrophage phenotypes, we speculate that the modulatory effect of blackcurrant polyphenols on macrophage phenotype is still valid at least in the intestine where macrophages are likely exposed to intact polyphenols. Importantly, intestinal macrophages are constantly renewed by Ly6C^hi^ monocytes, which differentiate into M1 macrophages, in response to low inflammatory signals from luminal contents and gut microbiota [36]. Furthermore, hyporesponsiveness to bacterial ligands is one of the characteristics of intestinal macrophages for gut homeostasis [37]. Therefore, the suppressive effect on M1 polarization of polyphenols in blackcurrant may be beneficial for obesity-associated inflammation in intestinal macrophages, which could affect inflammatory states at the whole body level.

## 5. Conclusions

In this present study, we demonstrated that BCE suppresses M1 polarization of macrophages, leading to repressed pro-inflammatory responses. Furthermore, this study suggests that metabolites of blackcurrant may not exert the anti-inflammatory effect of blackcurrant directly by altering macrophage phenotypes, but it may attenuate inflammatory responses in macrophages by modulating levels of obesity-induced circulating pro-inflammatory factors.
